# Rapid detection of pathogenic *E. coli* based on CRISPR Cas system

**DOI:** 10.3389/fmicb.2024.1423478

**Published:** 2024-06-26

**Authors:** Pallavi Rathore, Ashesh Basnet, Agnes Kilonzo-Nthenge, Korsi Dumenyo, Zeinab Yadegari, Ali Taheri

**Affiliations:** ^1^Department of Agricultural Science and Engineering, College of Agriculture, Tennessee State University, Nashville, TN, United States; ^2^Department of Food and Animal Sciences, College of Agriculture, Tennessee State University, Nashville, TN, United States; ^3^Department of Life and Physical Sciences, Fisk University, Nashville, TN, United States

**Keywords:** CRISPR, pathogenic, *E. coli*, rapid, sensitive, molecular detection

## Abstract

Access to safe and nutritious food is critical for maintaining life and supporting good health. Eating food that is contaminated with pathogens leads to serious diseases ranging from diarrhea to cancer. Many foodborne infections can cause long-term impairment or even death. Hence, early detection of foodborne pathogens such as pathogenic *Escherichia coli* strains is essential for public safety. Conventional methods for detecting these bacteria are based on culturing on selective media and following standard biochemical identification. Despite their accuracy, these methods are time-consuming. PCR-based detection of pathogens relies on sophisticated equipment and specialized technicians which are difficult to find in areas with limited resources. Whereas CRISPR technology is more specific and sensitive for identifying pathogenic bacteria because it employs programmable CRISPR-Cas systems that target particular DNA sequences, minimizing non-specific binding and cross-reactivity. In this project, a robust detection method based on CRISPR-Cas12a sensing was developed, which is rapid, sensitive and specific for detection of pathogenic *E. coli* isolates that were collected from the fecal samples from adult goats from 17 farms in Tennessee. Detection reaction contained amplified PCR products for the pathogenic regions, reporter probe, Cas12a enzyme, and crRNA specific to three pathogenic genes—stx1, stx2, and hlyA. The CRISPR reaction with the pathogenic bacteria emitted fluorescence when excited under UV light. To evaluate the detection sensitivity and specificity of this assay, its results were compared with PCR based detection assay. Both methods resulted in similar results for the same samples. This technique is very precise, highly sensitive, quick, cost effective, and easy to use, and can easily overcome the limitations of the present detection methods. This project can result in a versatile detection method that is easily adaptable for rapid response in the detection and surveillance of diseases that pose large-scale biosecurity threats to human health, and plant and animal production.

## Introduction

Millions of individuals are impacted by bacterial infections each year, making it a major global health challenge. The majority of these bacterial infections are food borne. Annually, 48 million cases of foodborne illness are reported in the USA which is equivalent to 1 in 6 people (Food Safety and Inspection Service, 2020). In addition, consuming contaminated food costs low- and middle-income nations US$ 110 billion annually in lost productivity and medical costs (World Health Organization, 2022). One of the most common bacterial causes of foodborne illness is pathogenic *Escherichia coli*. It is the leading cause of most foodborne illnesses in the United States and around the world (Manges and Johnson, 2012). *E. coli* is generally a beneficial bacterium that is crucial for the initial colonization of the human gut, especially in newborns. As a facultative anaerobe, it serves to provide a conducive environment for other anaerobic bacteria to grow in the gut mucosal surface by depleting oxygen. Moreover, the bacterium creates vitamin K, which is required for blood coagulation, as well as colonization resistance against harmful pathogens (Martinson and Walk, 2020). Most strains of *E. coli* are harmless and even beneficial, aiding in digestion and producing essential vitamins. However, certain strains, such as *E. coli* O157:H7, can cause foodborne illnesses when ingested through contaminated food or water. *E. coli* infections cause 265,000 illnesses and 100 fatalities in the United States each year (World Health Organization, 2022). Human diarrheagenic and extraintestinal *E. coli* strains are divided into nine pathovars. Among them, seven pathotypes of enteric pathogenic *E. coli* have been reported including, enteropathogenic *E. coli* (EPEC), enterotoxigenic *E. coli* (ETEC), Vero toxin-producing/Shiga toxin-producing *E. coli* (VTEC/STEC), which includes the well-known subgroup, enteroaggregative *E. coli* (EAEC), enterohaemorrhagic *E. coli* (EHEC), enteroinvasive *E. coli* (EIEC), and diffuse adherent *E.coli* (DAEC) (Jafari et al., 2012). Pathogenic *E. coli* strains possess various virulence factors and genes enabling them to cause diseases in humans. These include adhesion factors such as type 1 pili, P fimbriae, and S fimbriae, which facilitate colonization of epithelial cells. Enterotoxigenic *E. coli* (ETEC) strains produce heat-stable toxin (ST) and heat-labile toxin (LT), while enterohemorrhagic *E. coli* (EHEC) strains produce Shiga toxins (stx genes), leading to severe complications like bloody diarrhea and hemolytic uremic syndrome (HUS). Pathogenic strains may also harbor a type III secretion system (T3SS) for injecting bacterial effectors into host cells, along with iron acquisition systems and capsule formation genes for immune evasion. Additionally, antibiotic resistance genes further complicate treatment. Key genes associated with these factors include stx, eae, and hly genes, though the expression and combination of these factors vary among strains (Sora et al., 2021).

Among these strains, STEC is a major foodborne pathogen that causes serious sickness in humans all over the world. Farm animals like goats, sheep, cattle and bovine food supplies as well as fresh produce infected with animal’s excrement are the most prevalent origins of illness outbreaks in the United States. Isolates of STEC have two virulence genes including, hlyA gene and at least one of the stx genes (stx1 or stx2) which produce hemolysin and shiga-toxins, respectively, as virulence factors for this pathotype (Meng et al., 1998).

Multiple techniques have been developed for detecting *E. coli* in foods and clinical samples. Traditional detection method, such as media specific bacterial culture (Hariri, 2022) and polymerase chain reaction (PCR) (Choi et al., 2018), have long been the foundation of pathogen identification. However, these procedures have substantial limits in terms of time, non- specificity, time consuming, and labor intensive, which prevents widespread implementation, especially in resource-constrained situations. Furthermore, while enzyme-linked immunosorbent assay (ELISA)-like approaches provide great specificity, their implementation requires significant expertise, skilled technicians, and sophisticated equipment, limiting their accessibility and applicability in a variety of conditions (Shen et al., 2014).

Therefore, there is a need to develop rapid, precise, and low-cost detection methods for detection of such pathogens that can be accessible for most users especially in rural areas or developing nations.

These limitations can be addressed by using a recently developed gene editing tool called CRISPR (Clustered Regularly Interspaced Short Palindromic Repeats) as a detection technology for *E. coli* (Abudayyeh and Gootenberg, 2021; Selvam et al., 2022). The CRISPR/Cas-mediated detection method is a very rapid technology that can detect any DNA with great specificity and sensitivity. Compared to other methods, this approach is less expensive and less complicated as it only requires two main components including a Cas enzyme and a guide RNA (gRNA). It also does not require any expensive equipment and is versatile enough that can be adapted to different organisms by just changing gRNA that is specific to the new organism. As a result, CRISPR based detection methods are especially valuable for public health monitoring and response, where early diagnosis and action can avoid disease outbreaks (Chakraborty et al., 2022).

CRISPR systems are fundamentally an essential component of a microbial immune system which recognizes foreign nucleic acids based on their sequence and eliminates the invading pathogen via endonuclease activity linked with the CRISPR-associated (Cas) enzyme (Mojica et al., 2005). The Cas enzyme (CRISPR-associated protein) and a guide RNA (gRNA) are the two main components of the system. The guide RNA is designed to identify and attach to a specific DNA sequence in the target genome, after which the Cas enzyme cuts the DNA at that location. This method enables precise and effective DNA editing (Asmamaw and Zawdie, 2021). The CRISPR-Cas system has been used not only for genome and RNA editing, but also for nucleic acid detection in recent years and CRISPR/Cas-based technologies have just emerged with the potential to revolutionize the pathogen detection in various samples (Wang et al., 2020). CRISPR-Cas systems are majorly divided into two classes- class 1 and class 2 based on the architectures of their effector modules involved in crRNA processing and interference. Class 1 systems feature effector modules consisting of numerous Cas proteins, some of which form crRNA-binding complexes (such as the Cas complex in type I systems) that mediate pre-crRNA processing and interference with contributions from additional Cas proteins. Class 2 systems, on the other hand, have a single multidomain crRNA-binding protein (such as Cas9 in type II systems) that combines all functions necessary for interference and, in certain versions, those involved in pre-crRNA processing (Makarova et al., 2020). The most commonly used CRISPR based detection systems rely on the use of either these enzymes: Cas9, Cas12, Cas13, and Cas14 that are all members of the class 2 system (Wang et al., 2020). Class 2 CRISPR-Cas systems, particularly CRISPR-Cas9, Cas12a, and Cas13a, have been widely used in nucleic acid detection tests for human diseases because to their simplicity and high efficiency (Fapohunda et al., 2022).

While CRISPR has received much attention for its potential in gene editing in diverse species, its adaptability extends well beyond this one use. Recent research have demonstrated the usefulness of CRISPR-based approaches in identifying a wide range of pathogens, including viruses, fungi, and bacteria, emphasizing their broad relevance in infectious illness diagnosis (Kim et al., 2021).

For example, researchers have successfully developed CRISPR-based tests for the rapid identification of Salmonella species in food, water, and clinical specimens. These tests have great sensitivity and specificity, making them essential instruments for guaranteeing food safety and disease surveillance (Bugarel et al., 2018). Furthermore, CRISPR-based approaches have been investigated for the detection of Human Papillomavirus (HPV), a sexually transmitted virus linked to cervical cancer and other cancers (Xu et al., 2020). These approaches enable the quick and sensitive identification of HPV infection, allowing for early detection and action to avoid various human diseases. In addition, CRISPR technology has been used to detect viral diseases such as SARS-CoV-2, the cause of the COVID-19 pandemic (Rahimi et al., 2021). Researchers used the specificity and sensitivity of CRISPR-based assays to provide quick and accurate diagnostic tools to prevent COVID-19 transmission and guide public health interventions. These examples demonstrate CRISPR technology’s transformational potential in tackling a wide range of public health concerns faced by various diseases.

In this project, we designed specific crRNA based on pathogenic genes in *E. coli* (STEC) and reported their use in detection of pathogenic *E. coli* samples that were collected from different farms in Tennessee. This system is based on CRISPR-Cas12a sensing, which is rapid, highly sensitive and specific for detection of pathogenic *E. coli* isolated from fecal samples collected directly from goat farms in Tennessee because the presence of pathogenic *E. coli* in goat fecal samples is a significant concern due to its potential to cause foodborne illness in humans, as it can lead to cross-contamination in the farm environment and increase the risk of transferring bacteria onto carcasses during slaughtering processes, resulting in severe gastrointestinal infections.

## Materials and methods

### Study sites and fecal sample collection

**Figure 1:**
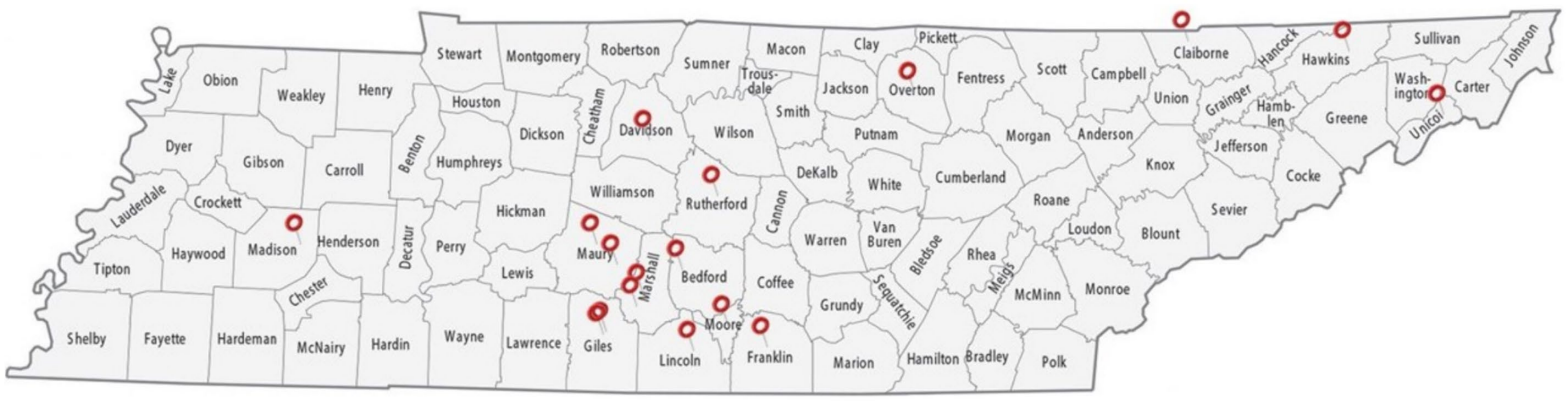
A map of the counties in Tennessee from which fecal samples were collected. Samples were obtained aseptically directly from live animals in 17 goat farms located in 14 different counties to acquire a diverse set of samples—Marshall, Giles, Bedford, Lincoln, Franklin, Rutherford, Davidson, Unicoi, Claiborne, Madison, Maury, Moore, Overton, and Hawkins.

The research was approved by the Institutional Review Board (IRB) and Institutional Animal Care and Use Committee (IACUC, #TSU21-04) of Tennessee State University (TSU). During the sample collection process, strict adherence to animal welfare and protection standards and guidelines was ensured. Fecal samples were aseptically collected from 17 goat farms in Tennessee (Figure 1). These farms were in 14 counties in Tennessee (Marshall, Giles, Bedford, Lincoln, Franklin, Rutherford, Davidson, Unicoi, Claiborne, Madison, Maury, Moore, Overton, and Hawkins).

The fecal samples from goats and sheep were collected following a modified (Bauman et al., 2016) method. Each animal’s rectum yielded 4–6 grams of sample, which were then placed in individual sterile plastic vials by the same handler. Disposable polyethylene gloves, lubricated with 0.3 mL of sterile OB Lube Non-Spermicidal Sterile Lubricating Jelly (VetOne, Boise, Idaho, United States), were used for each animal to prevent cross-contamination.

These samples were appropriately labeled. Transported to the Food Safety and Microbiology Laboratory at TSU in a cooler equipped with ice packs, and stored at −20°C for approximately 1 week before undergoing microbial analysis.

### Isolation and culturing of *E. coli* from fecal samples

The collected fecal samples were initially thawed to room temperature. Approximately 2 grams of each sample were then homogenized in 18 mL of Buffered Peptone Water (CM0509; Oxoid, Basingstoke, United Kingdom) within a sterile stomacher bag (Fisher Scientific, United States) at a speed of 230 rotations per minute (rpm). Subsequently, 10 mL of the homogenized solution from each sample was transferred to individual test tubes and incubated at 37°C for 18–24 h. These pre-enriched solutions were then transferred to Tryptic Soy Broth (TSB) (BD, Franklin Lakes, NJ) for enrichment. The TSB broth cultures were further incubated at 37°C for an additional 18 to 24 h. Following the incubation period, the overnight cultures were streaked onto selective agar plates, specifically Eosin Methylene Blue (BD, Franklin Lakes, NJ) agar plates, for *E. coli* selection. These plates were incubated at 37°C for another 18–24 h.

Presumptive single colonies, with a metallic green sheen, isolated from the agar plates were subsequently sub-cultured into TSB broth and incubated at 37°C for a further 18–24 h for DNA extraction and further confirmation by Polymerase Chain Reaction (PCR).

### DNA extraction from isolated *E. coli* colonies

DNA isolation from the overnight TSB broth cultures was carried out by extracting DNA from the presumed isolates using the Qiagen (Hilden, Germany) DNeasy^®^ UltraClean^®^ Microbial Kit, DNeasy^®^ UltraClean^®^ Microbial Kit (QIAGEN, Hilden, Germany), following the recommended procedures provided by the manufacturer. Approximately 2 mL of culture from each sample was used for DNA extraction, and at the conclusion of the process, 100 μL of DNA was eluted. The concentration of DNA in each sample was quantified using the Qubit 1× High Sensitivity dsDNA assay kit (Invitrogen, Waltham, Massachusetts, United States).

### Molecular confirmation by PCR

To confirm the identity of the presumptive bacterial isolates, a molecular confirmation step was undertaken using Polymerase Chain Reaction (PCR). The PCR mixture was prepared following the manufacturer’s instructions for Qiagen HotStar Taq Polymerase (Germantown, Maryland, United States). Each PCR reaction used approximately 100 nanograms per milliliter (ng/mL) of DNA, as determined by prior DNA concentration measurements. The PCR mixture was composed of the following components: 10 μL of 2X HotStar Taq Plus Master Mix, 0.5 μM of both forward and reverse primers, 2 μL of 10X CoralLoad Concentrate dye, 100 ng of template DNA, and RNase-free water was added to achieve a final reaction volume of 20 μL. Forward (AGA GTT TGA TCA TGG CTC AG) and reverse primers (GGA CTA CCA GGG TAT CTA AT) for 16S rRNA genes was used for the PCR confirmation per (Holland et al., 2000). PCR protocol included an initial denaturation step at 94°C for 5 min, followed by 32 cycles consisting of 94°C for 1 min, an annealing step at 58°C for 30 s, and an extension at 72°C for 1 min. Finally, a concluding elongation step was performed at 72°C for 10 min.

**Figure 2:**
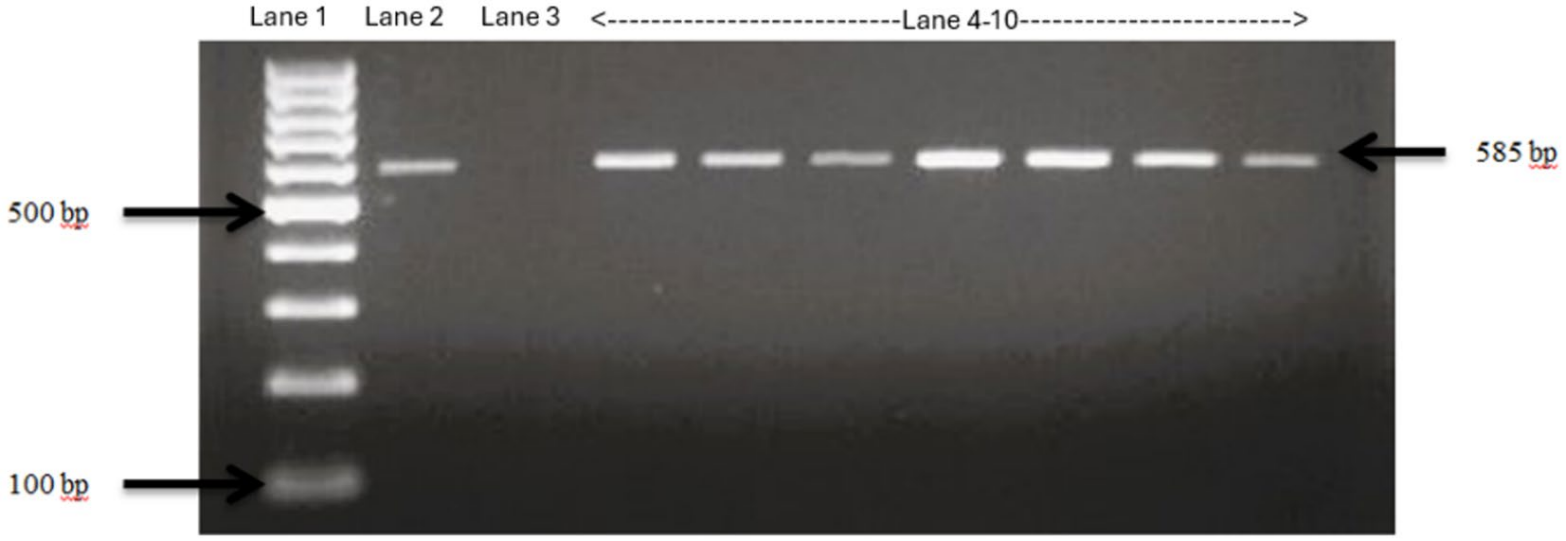
Confirmation of *E. Coli* isolates using specific primers. Lane 1: 100 bp ladder, Lane 2: positive control *E. Coli* ATCC 25922, Lane 3: negative control *Salmonella typhimurium*, Lane 4–10: fecal samples collected from Tennessee farms which show bands same as the positive control, confirming the isolates as *E. Coli*.

The PCR product was run in a 2% agarose gel at 90 V for 1.5 h. The agarose gel was finally viewed under Bio-Doc Gel Doc Ez Imager Documentation Imaging Electrophoresis System (BioRad, Hercules, California) and positive isolates were confirmed (Figure 2).

### Target genes and gRNA design

This study is focused on detection of Enterohemorrhagic *Escherichia coli* (EHEC) that are a major group of food-borne pathogens. These strains are defined by presence of specific virulence factors including: hlyA gene (NP_052624.1) that is an EHEC-specific plasmid encoded hemolysin; at least one Shiga-like toxin [encoded by stx1(AB015056.1) or stx2(NP_309232.1)]; most of these strains also produce intimin that is encoded by eaeA (NP_312586.1). DNA sequence information for these genes were retrieved from NCBI and submitted to the Cas-Designer tool at rgenome.net for designing specific gRNA for Cas9 detection. We then used Benchling tool to design specific primers that are spanning these gRNAs.

#### PCR confirmation of virulence genes

**Figure 3:**
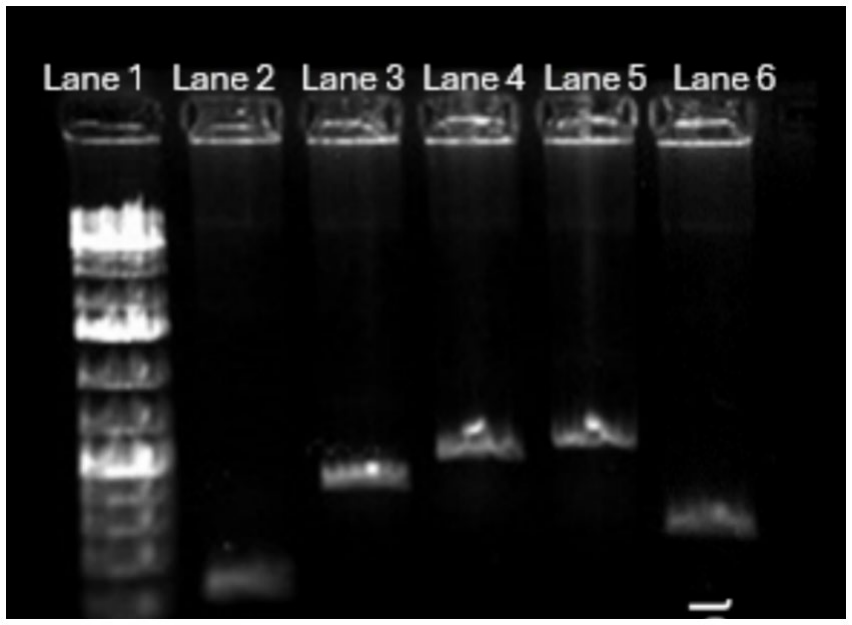
PCR amplification of four virulence genes in pathogenic *E. coli* with positive control. The positive control is *E. coli* DNA amplified with appropriate 16S rRNA primers. Lane 1—1 kb Ladder, Lane 2—stx2 gene amplified at 200 bp, Lane 3—Stx1 gene amplified at 500 bp, Lane 4—HlyA gene amplified at 900 bp, Lane 5—eaeA gene amplified at 900 bp, Lane 6—positive control amplified at 400 bp.

All the four virulence genes (stx1, stx2, hlyA, and eaeA) were amplified (Figure 3) using gene specific primers (Table 1) and then the PCR product was used for CRISPR reactions. A positive pathogenic sample that had all these 4 genes was used as control.

**Table 1:** Primers used for amplification of different virulence genes with their *T*_m_.

Virulence genes	Primer sequence (5′ to 3′)	*T*_m_	Product length
Stx1 gene	Forward-GTGGCACAGGGGATAATTTG	60	500 bp
	Reverse-AGAACGCCCACTGAGATCAT	60	
Stx2 gene	Forward-CCATGACAACGGACAGCAGTT	63	200 bp
	Reverse-CCTGTCAACTGAGCAGCACTTTG	65	
hlyA gene	Forward-TCTGCGGGAGTTAGTGCAGCCT	68	900 bp
	Reverse-CTTCACGTGACCATACATAT	51	

### CRISPR-based detection reactions

Three μL of PCR products for various virulence genes were combined with Cas12a enzyme, gene-specific crRNA, and fluorescent reporters and were kept in 37°C for 1 h (Table 2). First the CRISPR reactions were confirmed on the pathogenic *E.coli* (positive control) by setting six reactions on same sample. Out of which only one of the reactions had all the components and the rest had at least one of the component missing (Table 3).

**Table 2:** Components of CRISPR reaction and their concentrations used in this study.

Components	Amount
Lb Cas12a	0.5uL (1 μM)
crRNA	0.5uL (10 μM)
FAM/HEX reporter	1uL (10 μM)
PCR product	3 μL
Buffer 3	2 μL
Water	13 μL

**Table 3:** Six different reactions on the positive control with only reaction number 3 with all the components.

Reactions	1	2	3	4	5	6
PCR product	+	+	+	−	−	−
FAM/HEX reporter	+	+	+	+	+	+
Cas12a	−	+	+	−	+	+
crRNA	+	−	+	+	−	+

**Figure 4:**
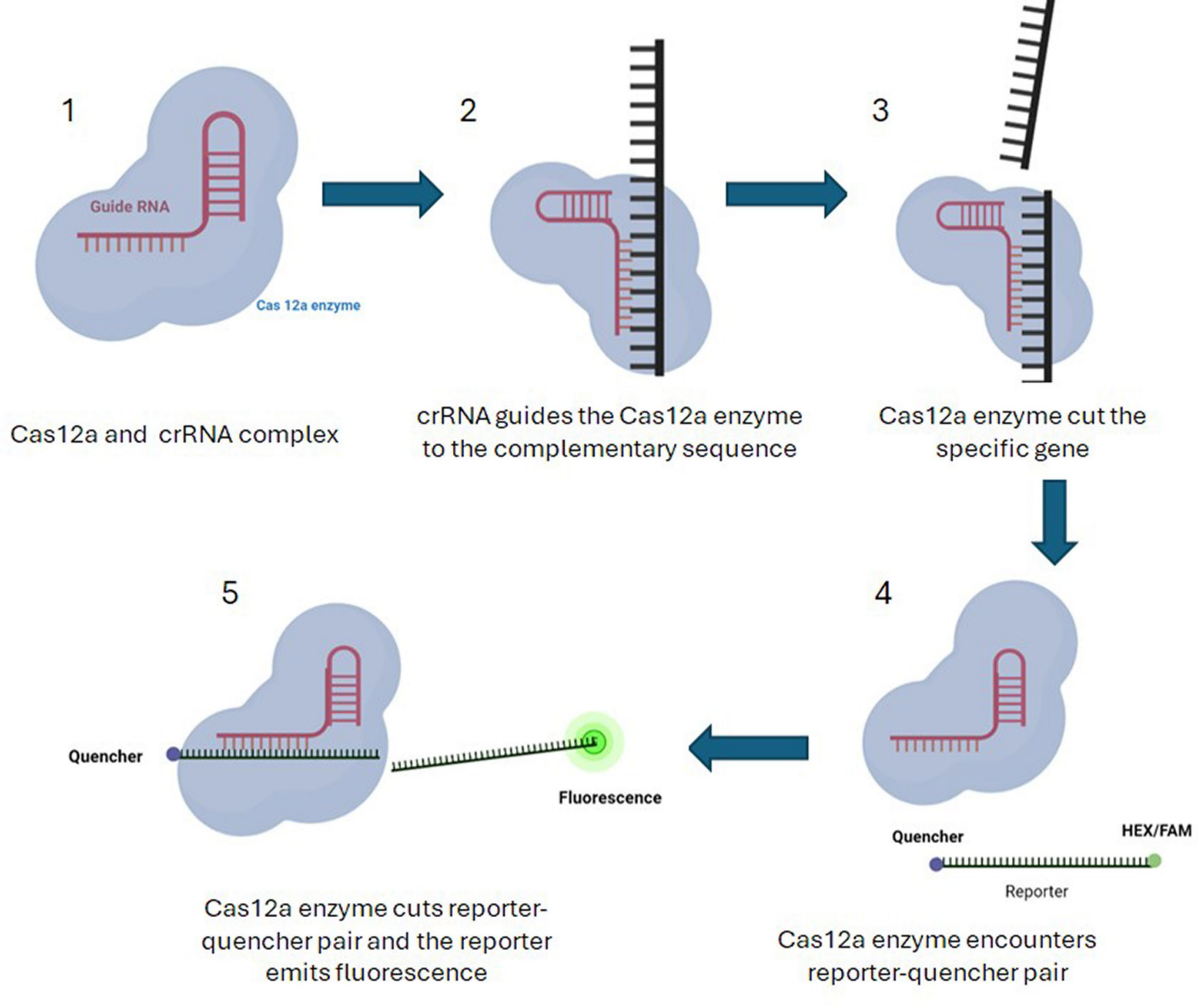
Pathogen detection using Cas12a along with HEX/FAM reporters coupled to a quencher. 1—Cas12a and crRNA complex is formed. 2—crRNA takes the complex to the sequence of the target virulence gene. 3—If the virulence gene is present in a sample, Cas12a and crRNA complex will detect and cut the complementary gene. 4—The Cas complex becomes active and proceeds to cut any single stranded nucleotide that is present in the reaction including reporter–quencher pairs. 5—When the reporter is separated from the quencher, it begins to emit fluorescence light after excitation under UV light. The presence of fluorescence light in the sample indicates the presence of targeted virulence gene in that sample.

**Figure 5:**
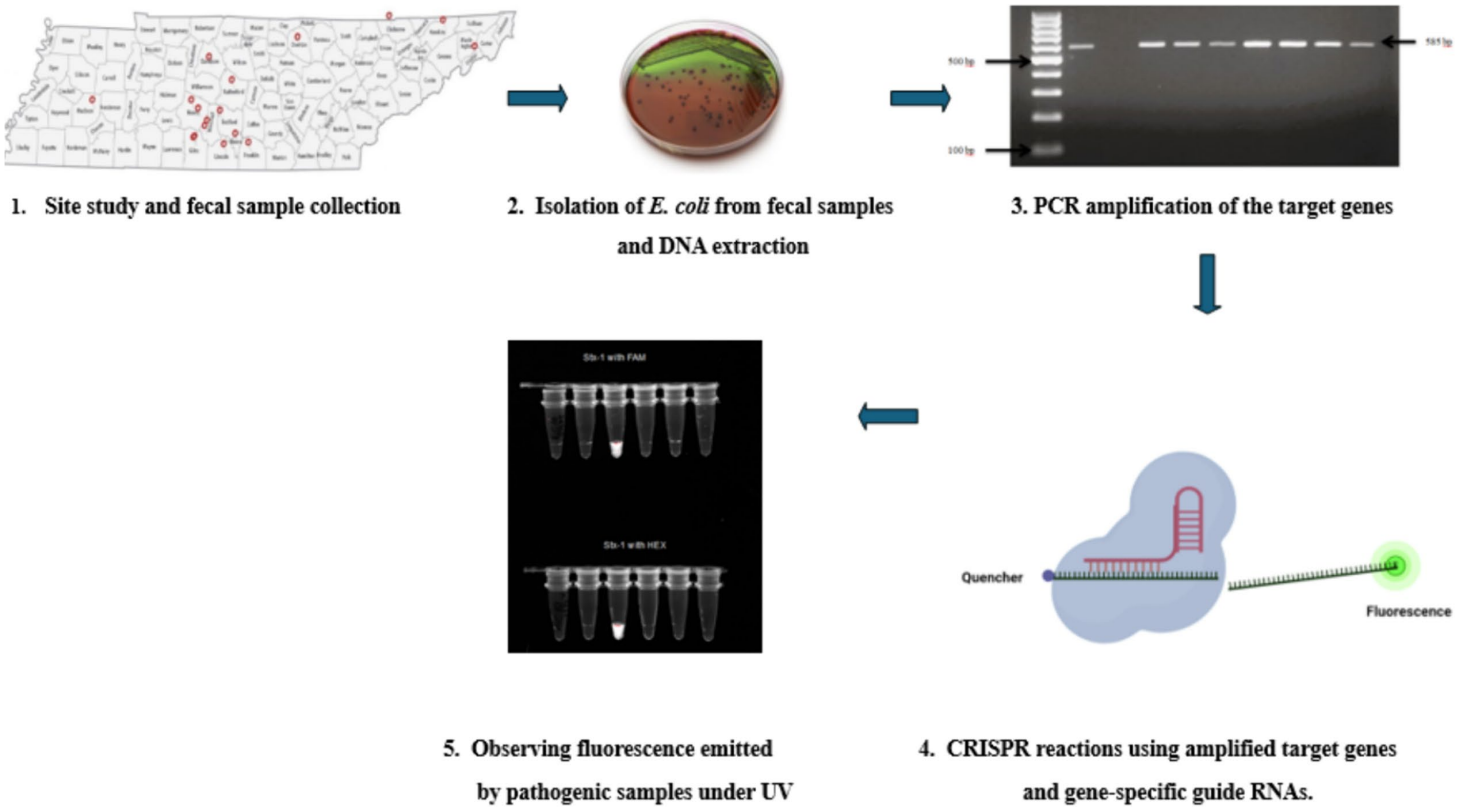
A flow chart showing the whole process—1. Studying the sites and collection of fecal samples directly from goats from 14 counties in Tennessee. 2. Isolation *E. Coli* colonies by culturing them on Eosin Methylene Blue agar plates and then extracting DNA from them using the Qiagen (Hilden, Germany) DNeasy^®^ UltraClean^®^ Microbial Kit, DNeasy^®^ UltraClean^®^ Microbial Kit (QIAGEN, Hilden, Germany). 3. Amplification of the four virulence genes (stx1, stx2, hlyA, and eaeA) using gene-specific primers. 4. Using amplified virulence genes combined with Cas12a enzyme, gene-specific crRNA, and fluorescent reporters for CRISPR reactions at 37°C for 1 h. 5. Observing the Fluorescent light emitted by pathogenic samples under UV light.

HEX/FAM reporters (Table 4) coupled to a quencher were used to select *E. coli* samples containing virulence genes (pathogenic *E.coli*). If the virulence gene is present in a sample, Cas12a and crRNA (Table 5) complex will detect and cut the complementary gene. The Cas complex becomes active and proceeds to cut any single stranded nucleotide that is present in the reaction including reporter–quencher pairs. When the reporter is separated from the quencher, it begins to emit fluorescence light after excitation under UV light. The presence of fluorescence light in the sample indicates the presence of targeted virulence gene in that sample (Figures 4, 5).

**Table 4:** Sequence for the HEX and FAM reporters.

Reporters	Sequence
HEX	5′-/5HEX/TTA TTA TT/3IABkFQ/-3’
FAM	5′-/56-FAM/TTA TT/3IABkFQ/-3’

**Table 5:** Sequence for the crRNA designed for different genes.

Virulence genes	Sequence for crRNA
Stx1	rUrArArUrUrUrCrUrArCrUrCrUrUrGrUrArGrArUrCrArGrGrUrArCrArArCrArGrCrGrGrUrUrArCrArUrUrG
Stx2	rUrA rArUrU rUrCrU rArCrU rArArG rUrGrU rArGrA rUrArA rUrGrG rGrUrA rCrUrG rUrGrC rCrUrG rUrUrA rCrUrG
HlyA	rUrA rArUrU rUrCrU rArCrU rArArG rUrGrU rArGrA rUrArU rCrCrU rCrArA rArArA rGrGrG rArCrC rArUrA rGrArC

## Results

### Molecular confirmation by PCR

**Figure 6:**
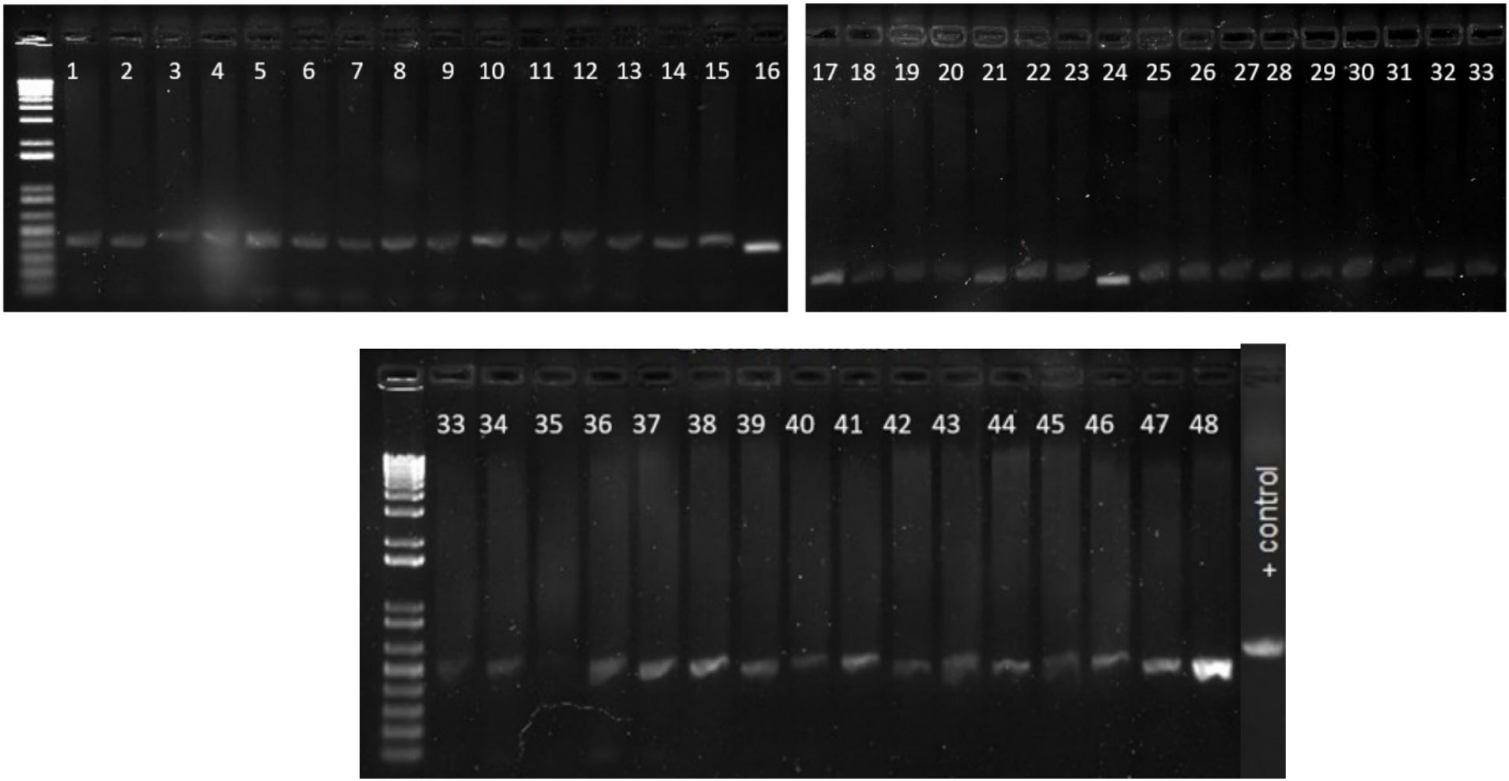
Gel picture of the PCR results for confirmation of *E. coli* using specific primers. The PCR results consistently revealed the anticipated 500 bp fragment across all samples indicating that all the collected samples are indeed *E. coli* isolates. Lane 1–48 *E. Coli* isolates, Positive control *E. coli* ATCC 25922.

Identity of all the samples used in the study were confirmed using two *E.coli* confirmation methods including media selection and PCR assay. These samples were then checked for presence of different virulence genes discussed above and were later used for pathogen detection in CRISPR assay. The PCR results consistently revealed the anticipated 500 bp fragment across all samples indicating that all the collected samples are indeed *E. coli* isolates (Figure 6).

### PCR confirmation of virulence genes

PCR reactions were carried out for 49 *E.coli* samples as well as one positive control using primers designed for four virulence genes in STEC *E. coli* (stx1, stx2, hlyA, and eaeA). All the samples showed positive PCR results for stx1 gene (Figure 6). Similarly, 4 and 6 samples showed amplification for stx2 (Supplementary Figure S1) and hlyA (Supplementary Figure S2), respectively.

### CRISPR-based detection reactions

**Figure 7:**
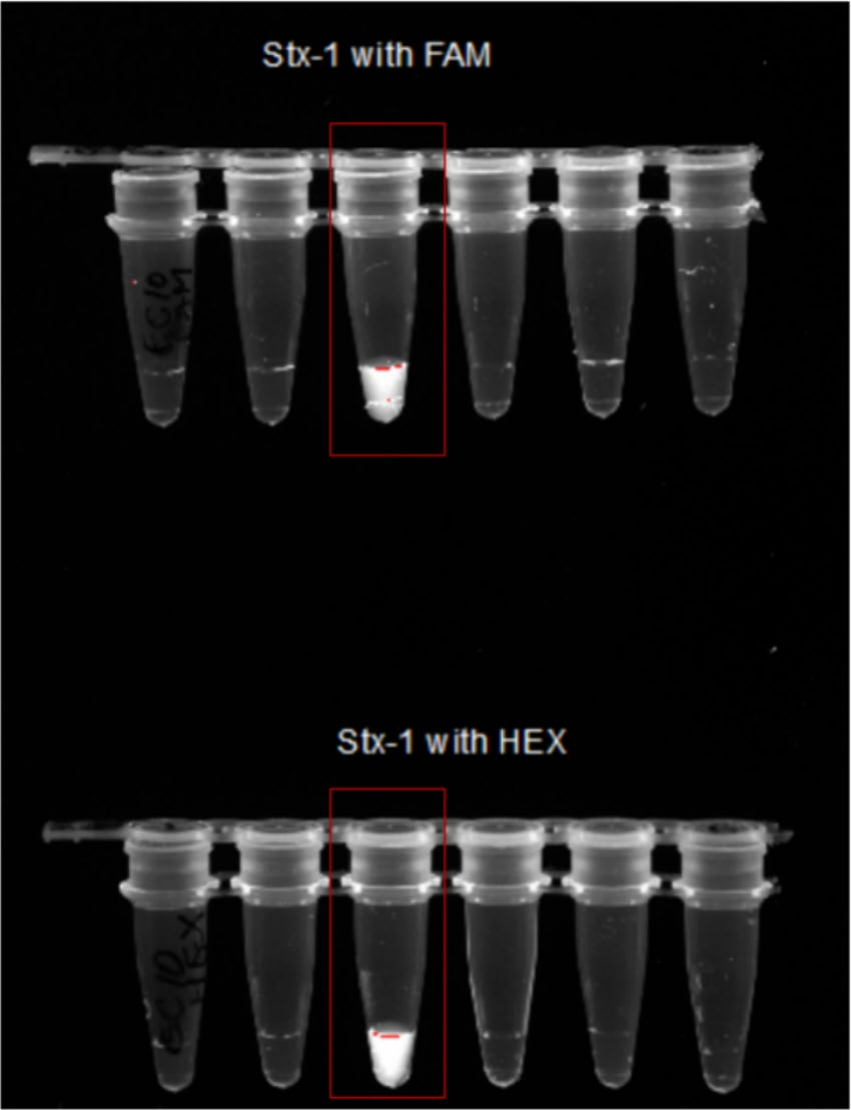
Confirmation of Stx1 CRISPR detection using both FAM and HEX probes. Image showing only reaction number 3 with all the components as discussed above-emitted fluorescence under the UV light. This shows that only reaction with all components—Lb Cas12a, crRNA, FAM/HEX reporter, PCR product, Buffer 3, and Water will emit the florescent light.

**Figure 8:**
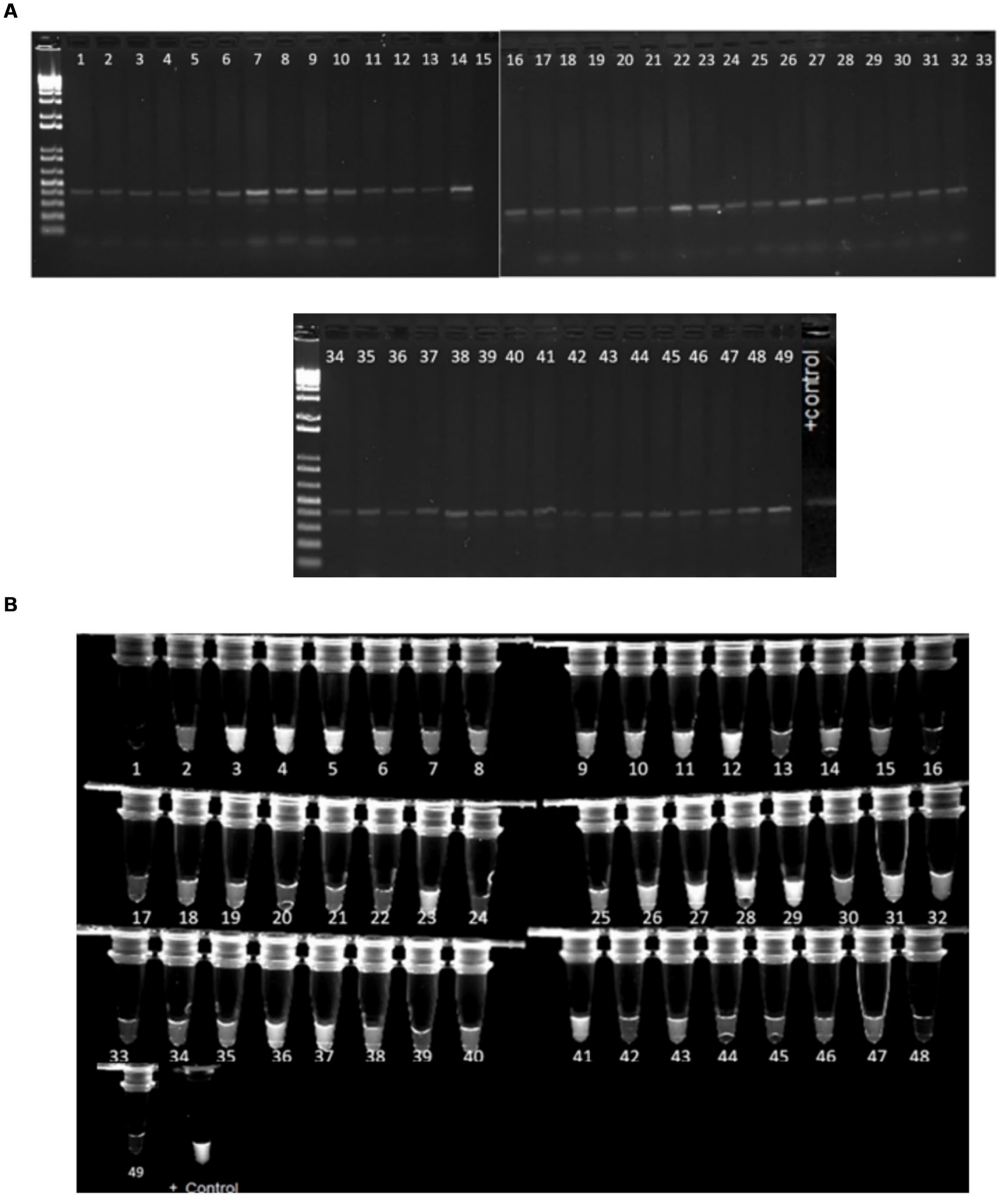
**(A)** Gel image of samples with a 500 bp band size, suggesting positive PCR findings for stx1 gene amplification. **(B)** Image of samples with the stx1 virulence gene generating fluorescence under UV light after the CRISPR response. The positive control is *E. coli* O157. Approximately 45 samples out of 49 that displayed bands after PCR amplification also fluoresced under UV light following CRISPR responses.

The crRNAs ability to recognize target genes were confirmed using a pathogenic *E. coli* sample (*Escherichia coli* O157:H7). Only the reaction with all the components emitted fluorescence light under the UV exposure (Figure 7). 1 μL of the PCR products from each virulence gene reaction were used in subsequent CRISPR reactions to detect the presence of the gene as mentioned earlier. When exposed to UV, 92% of samples that had a positive PCR result displayed fluorescence following the CRISPR response for all three genes-stx1 (Figure 8), stx2 (Supplementary Figure S1), and hlyA (Supplementary Figure S2) as predicted (Table 6).

**Table 6:** Summary of key findings.

Category	Description	Results
*E. Coli* confirmation	Confirmation of *E. coli* identity in all samples using media selection and PCR assay	All samples showed the anticipated 500 bp fragment, confirming *E. coli* identity
Virulence GeneAmplification	PCR amplification of four virulence genes (stx1, stx2, hlyA, eaeA) in *E. coli* samples	stx1: Positive in all samples stx2: Positive in 4 samples hlyA: Positive in 6 samples
CRISPR-based detection	Detection of virulence genes using CRISPR reactions, confirmed by fluorescence under UV light	stx1: 92% of PCR-positive samples showed fluorescence stx2: 2 out of 3 PCR-positive samples showed fluorescence hlyA: 4 out of 7 PCR-positive samples showed fluorescence

## Discussion

A robust detection method for the rapid and specific detection of pathogenic *Escherichia coli* (*E. coli*) using CRISPR-Cas12a sensing can help to access safe and nutritious food for maintaining good health, and avoid food contaminated with pathogens, including pathogenic *E. coli*, which can lead to severe diseases and even death. Therefore, the development of efficient and reliable detection methods is essential for public safety.

Conventional methods for detecting pathogenic bacteria are challenging to implement in resource-limited areas. To address these limitations, we employed CRISPR-Cas12a sensing as a detection platform for pathogenic *E. coli*. This technique combines the advantages of PCR amplification and CRISPR-based detection, offering a rapid, highly sensitive, and specific method for pathogen identification. Our detection reaction consisted of amplified PCR products targeting the pathogenic regions, a reporter probe, Cas12a enzyme, and crRNA specific to three pathogenic genes (stx1, stx2, and hlyA). The PCR primers used for amplification of these genes were based on previous reports and they were generating single bands in control and positive samples. However, in case of hlyA, we observed multiple amplifications in samples that were collected from Tennessee farms even at higher annealing temperatures. This indicates that optimization of PCR and design of better PCR primers is necessary for amplification of this gene.

The CRISPR reaction exhibited fluorescence when exposed to UV radiation, indicating the presence of pathogenic *E.coli*. This fluorescence-based detection method demonstrated high precision, sensitivity, and speed, surpassing the limitations of current detection approaches. Furthermore, it is cost-effective and relatively simple to use, which makes it accessible even in resource-limited settings where skilled technicians and advanced laboratory infrastructure may be scarce. Despite the promising results and advantages of our developed method, there are some limitations that should be acknowledged. One limitation is the requirement for PCR amplification as a preliminary step. While this step enhances the sensitivity of the assay, it adds an additional level of complexity to the overall process. Future studies could explore the integration of isothermal amplification techniques like LAMP PCR to simplify the workflow.

The development of a versatile detection method, such as the one presented in this study, holds significant implications for rapid response in disease detection and surveillance. It can effectively address large-scale biosecurity threats to human health, as well as plant and animal production. By quickly identifying and monitoring the presence of pathogenic *E. coli*, outbreaks can be contained, and appropriate preventive measures can be implemented to ensure public safety. Moreover, this technology can be used to make biosensors which can be used for rapid CRISPR testing in the field by replacing the PCR with LAMP PCR. This will completely remove the need for a lab for *E.coli* testing in the food samples.

We used amplified PCR products from samples collected which still rely on the use of sophisticated methods and instruments. This step can be avoided by switching to biosensor based detection systems that does not require PCR amplification and can also shorten the detection time to under 15 min (Ban et al., 2023).

## Conclusion

In conclusion, the development of this CRISPR-Cas12a sensing method for the detection of pathogenic *E. coli* represents a significant advancement in the field of foodborne pathogen detection. In this study we identified crRNAs that can be used for screening pathogenic *E. coli* from different samples. Out of the 49 *E. coli* samples examined in this study, 45 (92%) tested positive for the stx1 gene, 2 (4%) for the stx2 gene, and 4 (8%) for the hlyA gene, demonstrating varying prevalence rates among the studied genes. Further improvements can be made to shorten the time and complexity of the assay by switching to biosensor detection assays using the crRNAs that are reported in this study. Understanding the prevalence and distribution of pathogenic genes like stx1, stx2, and hlyA among *E. coli* isolates is crucial for evaluating the potential virulence and assessing the public health risks associated with *E. coli* contamination in goat farming and food production. The limited sample size of 49 is due to resource constraints; however, significant efforts were made to collect a diverse range of samples by traveling across various parts of the state and collaborating with small-scale farmers. Future research with a bigger sample size will assist in establishing the approach’s sensitivity and specificity.

While this technology is a fundamental approach, its key contribution is in identifying optimum guide RNAs for *E. coli* pathogenic factors. This foundation is intended to pave the way for the production of more rapid and sensitive detecting sensors in further research (Kaminski et al., 2021).

## Data availability statement

The datasets presented in this study can be found in online repositories. The names of the repository/repositories and accession number(s) can be found in the article/Supplementary material.

## Author contributions

PR: Data curation, Formal analysis, Investigation, Methodology, Writing – original draft, Writing – review & editing. AB: Data curation, Resources, Writing – original draft. AK-N: Conceptualization, Data curation, Methodology, Project administration, Resources, Supervision, Writing – original draft, Writing – review & editing. KD: Investigation, Methodology, Project administration, Resources, Supervision, Writing – original draft. ZY: Conceptualization, Methodology, Software, Writing – review & editing. AT: Conceptualization, Funding acquisition, Investigation, Project administration, Resources, Supervision, Visualization, Writing – original draft, Writing – review & editing.
