# Supplementary material for: Rapid detection of pathogenic E. coli based on CRISPR Cas system
Source: Front Microbiol. 2024 Jun 26;15:1423478. doi: 10.3389/fmicb.2024.1423478 (PMC11233538; doi:10.3389/fmicb.2024.1423478)
Supplement: Supplementary file 1 [file Table_1.DOCX]

**Supplementary**


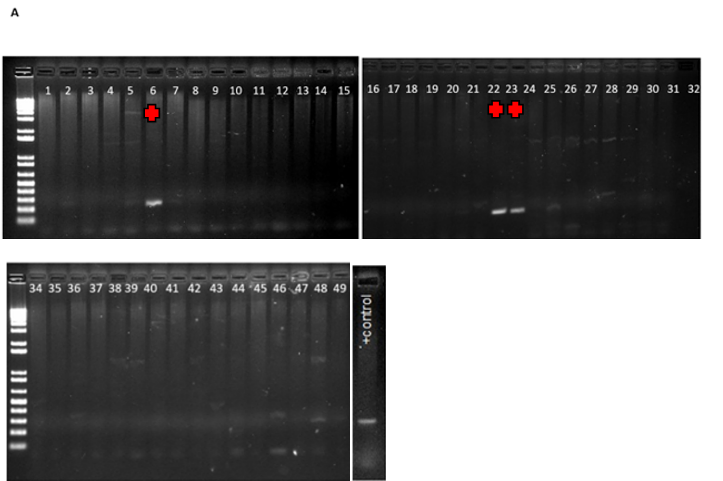


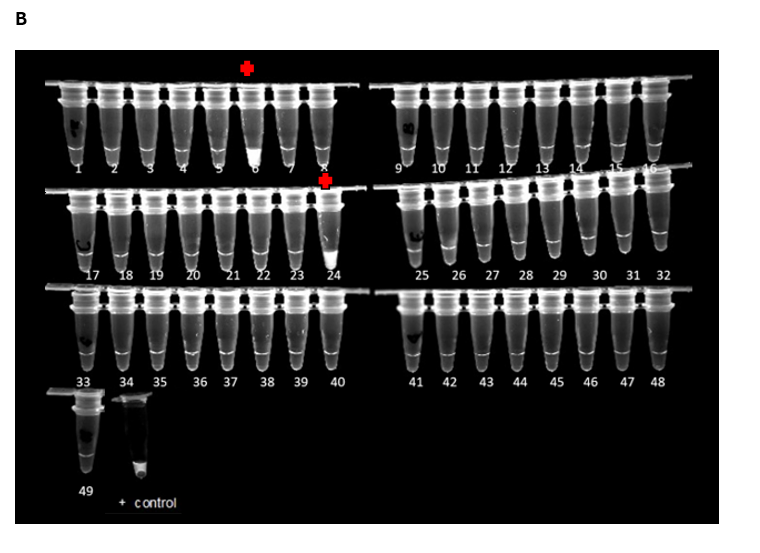


Figure 1 (A ) Gel image showing samples with positive PCR results for stx2 gene amplification.(B) Image showing samples with stx2 virulence gene emitting fluorescence under the UV light after the CRISPR reaction. The positive control is E. coli O157. Approximately 2 samples out of 3 that displayed bands after PCR amplification also fluoresced under UV light following CRISPR responses.


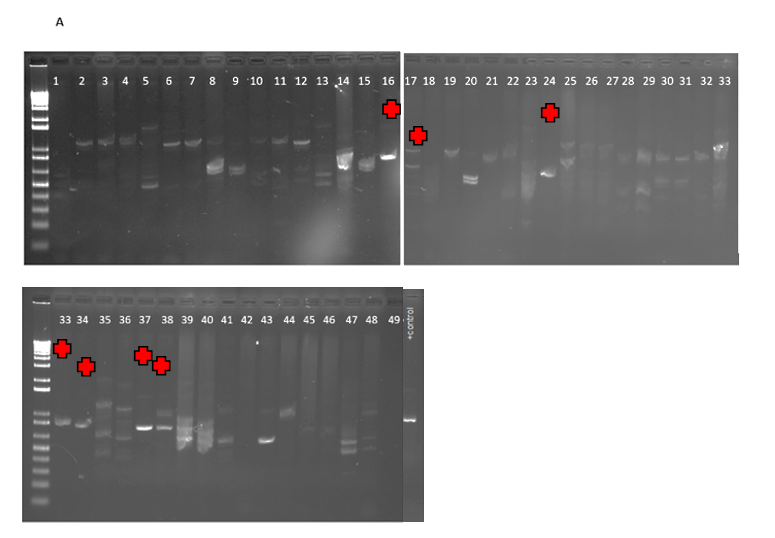


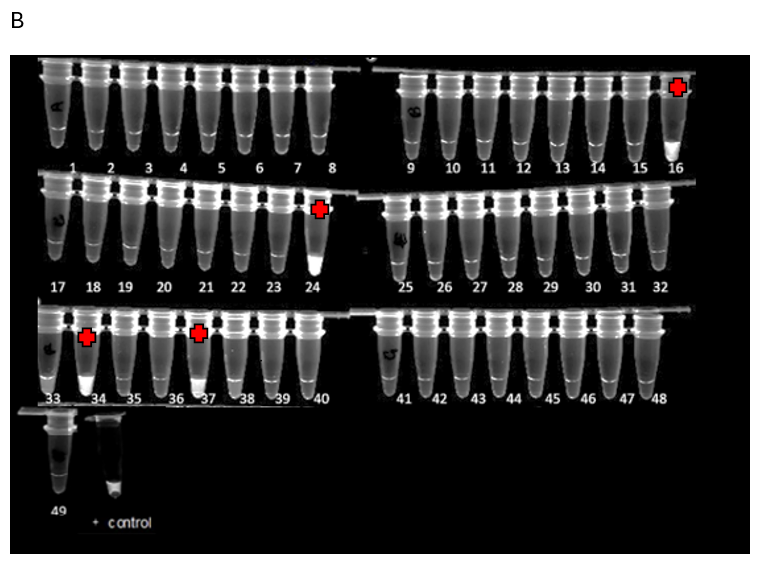


Figure 2 (A ) PCR amplification results for hlyA gene in 49 samples collected from Tennessee farms..(B) Image showing samples with HlyA virulence gene emitting fluorescence under the UV light after the CRISPR reaction. The positive control is E. coli O157. Approximately 4 samples out of 7 that displayed bands after PCR amplification also fluoresced under UV light following CRISPR responses.
